# Genetic and Functional Diversification of Small RNA Pathways in Plants

**DOI:** 10.1371/journal.pbio.0020104

**Published:** 2004-02-24

**Authors:** Zhixin Xie, Lisa K Johansen, Adam M Gustafson, Kristin D Kasschau, Andrew D Lellis, Daniel Zilberman, Steven E Jacobsen, James C Carrington

**Affiliations:** **1**Center for Gene Research and Biotechnology and Department of Botany and Plant Pathology, Oregon State UniversityCorvallis, OregonUnited States of America; **2**Department of Molecular, Celland Developmental Biology, University of California, Los Angeles, Los Angeles, CaliforniaUnited States of America; **3**Molecular Biology Institute, University of CaliforniaLos Angeles, Los Angeles, CaliforniaUnited States of America

## Abstract

Multicellular eukaryotes produce small RNA molecules (approximately 21–24 nucleotides) of two general types, microRNA (miRNA) and short interfering RNA (siRNA). They collectively function as sequence-specific guides to silence or regulate genes, transposons, and viruses and to modify chromatin and genome structure. Formation or activity of small RNAs requires factors belonging to gene families that encode DICER (or DICER-LIKE [DCL]) and ARGONAUTE proteins and, in the case of some siRNAs, RNA-dependent RNA polymerase (RDR) proteins. Unlike many animals, plants encode multiple DCL and RDR proteins. Using a series of insertion mutants of Arabidopsis thaliana, unique functions for three DCL proteins in miRNA (DCL1), endogenous siRNA (DCL3), and viral siRNA (DCL2) biogenesis were identified. One RDR protein (RDR2) was required for all endogenous siRNAs analyzed. The loss of endogenous siRNA in *dcl3* and *rdr2* mutants was associated with loss of heterochromatic marks and increased transcript accumulation at some loci. Defects in siRNA-generation activity in response to turnip crinkle virus in *dcl2* mutant plants correlated with increased virus susceptibility. We conclude that proliferation and diversification of *DCL* and *RDR* genes during evolution of plants contributed to specialization of small RNA-directed pathways for development, chromatin structure, and defense.

## Abstract 2

In plants, RNA-mediated silencing pathways have diversified in unique ways. This study elucidates the specific functions of some of the key regulators in development, chromatin structure, and pathogen defense

## Introduction

Eukaryotic small RNAs of approximately 21–24 nucleotides function as guide molecules in a remarkably wide range of biological processes, including developmental timing and patterning, formation of heterochromatin, genome rearrangement, and antiviral defense (Carrington and Ambros 2003; Finnegan and Matzke 2003; Lai 2003). They belong to at least two general classes, microRNA (miRNA) and short interfering RNA (siRNA). miRNAs (approximately 21–22 nucleotides) are found in plants and animals and are often phylogenically conserved within their respective kingdoms. They arise from non-protein-coding genes through formation of a precursor transcript followed by one or more nucleolytic processing steps (Lai 2003). Part of the precursor adopts a fold-back structure that interacts with a multidomain RNaseIII-like enzyme termed DICER or DICER-LIKE (DCL1 in *Arabidopsis*), which catalyzes accurate excision of the mature miRNA (Denli and Hannon 2003). The miRNAs then associate with ribonucleoprotein complexes that function to negatively regulate target genes controlling a range of developmental events, such as timing of cell fate decisions, stem cell maintenance, apoptosis, organ morphogenesis and identity, and polarity (Ambros 2003; Carrington and Ambros 2003).

siRNAs are chemically similar to miRNAs, although in plants they typically range in size between 21 and 24 nucleotides (Hamilton et al. 2002; Llave et al. 2002a; Tang et al. 2003). They are associated with both post-transcriptional forms of RNA interference (RNAi) and transcriptional silencing involving chromatin modification (Finnegan and Matzke 2003). siRNAs are processed from precursors containing extensive or exclusive double-stranded RNA (dsRNA) structure, such as transcripts containing inverted repeats or intermediates formed during RNA virus replication (Hannon 2002). siRNA precursors can also be formed by the activity of one or more cellular RNA-dependent RNA polymerases (RdRp), as was shown genetically in several screens for RNA silencing-defective mutants (Cogoni and Macino 1999; Dalmay et al. 2000; Mourrain et al. 2000; Smardon et al. 2000; Volpe et al. 2002). *Arabidopsis* plants contain at least three active RdRp genes, termed *RDR1*, *RDR2*, and *RDR6* (also known as *SDE1/SGS2*) (Dalmay et al. 2000; Mourrain et al. 2000; Yu et al. 2003). *RDR6* is necessary for sense transgene-mediated RNAi, but not for silencing of constructs that encode transcripts with hairpins containing extensive dsRNA structure (Dalmay et al. 2000; Mourrain et al. 2000; Beclin et al. 2002). In many animals, both miRNAs and siRNAs are formed by the activity of the same DICER enzyme (Grishok et al. 2001; Hutvágner et al. 2001; Ketting et al. 2001; Knight and Bass 2001; Provost et al. 2002; Zhang et al. 2002; Myers et al. 2003), although in plants they are formed by distinct DCL activities (Finnegan et al. 2003). *Arabidopsis* contains four *DCL* genes (*DCL1* to *DCL4*), only one of which (*DCL1*) has been assigned a definitive function in small RNA biogenesis (Park et al. 2002; Reinhart et al. 2002; Schauer et al. 2002). Biochemical data indicate, however, that multiple DCL activities or pathways catalyze formation of siRNAs of small-sized (approximately 21 nucleotides) and large-sized (approximately 24 nucleotides) classes (Tang et al. 2003). Endogenous siRNAs in plants arise from many types of retroelements and transposons, other highly repeated sequences, pseudogenes, intergenic regions (IGRs), and a few expressed genes (Hamilton et al. 2002; Llave et al. 2002a; Mette et al. 2002). Exogenous siRNAs can arise from both sense and hairpin transcript-forming transgenes and by viruses (Hamilton and Baulcombe 1999; Mette et al. 2000).

Both siRNAs and miRNAs function post-transcriptionally to suppress or inactivate target RNAs. siRNAs guide sequence-specific nucleolytic activity of the RNA-induced silencing complex (RISC) to complementary target sequences (Hannon 2002). Among other proteins, RISCs contain ARGONAUTE (AGO) family members that likely bind siRNAs or target sequences (Carmell et al. 2002). In plants and insects, post-transcriptional RNAi serves as an adaptive antiviral defense response (Waterhouse et al. 2001; Li et al. 2002). miRNAs are fully competent to guide nucleolytic function of RISC, provided that a target sequence with sufficient complementarity is available (Hutvágner and Zamore 2002; Doench et al. 2003; Tang et al. 2003). Many plant miRNAs function as negative regulators through this cleavage-type mechanism (Llave et al. 2002b; Rhoades et al. 2002; Emery et al. 2003; Kasschau et al. 2003; Palatnik et al. 2003; Tang et al. 2003; Xie et al. 2003). In animals, the level of complementarity between target and miRNA sequences is generally low, which inhibits nucleolytic activity. Animal miRNAs suppress translation of target mRNAs (Olsen and Ambros 1999; Reinhart et al. 2000). Some plant miRNAs may also function as translational suppressors (Aukerman and Sakai 2003; Chen 2003).

siRNAs also guide chromatin-based events that result in transcriptional silencing. Two lines of evidence support this view. First, in Schizosaccharomyces pombe and *Arabidopsis*, endogenous siRNAs from repeated sequences corresponding to centromeres, transposons, and retroelements are relatively abundant (Llave et al. 2002a; Mette et al. 2002; Reinhart and Bartel 2002). RNAi-related factors (DICER, RdRp, and AGO proteins) are required to maintain S. pombe centromeric repeats and nearby sequences in a transcriptionally inactive, heterochromatic state (Hall et al. 2002; Volpe et al. 2002). Mutants that lose RNAi component activities lose heterochromatic marks, such as histone H3 methylation at the K9 position (H3K9), as well as centromere function (Hall et al. 2002; Volpe et al. 2002, 2003). In plants, AGO4 is necessary to maintain transcriptionally silent epialleles of *SUPERMAN*. The *ago4* mutants lose both cytosine methylation, particularly at non-CpG positions, and H3K9 methylation at *SUPERMAN* and other constitutive heterochromatic sites (the Arabidopsis thaliana short interspersed element 1 [*AtSN1*] locus) (Zilberman et al. 2003). And, second, heterochromatin formation of nuclear DNA can be triggered, in a sequence-specific manner, by post-transcriptional silencing of cytoplasmic RNAs (Jones et al. 1999; Aufsatz et al. 2002; Schramke and Allshire 2003). The RNA-directed DNA methylation (RdDM) signal transmitted from the cytoplasm to the nucleus is most likely siRNA. The prevailing view states that chromatin-based silencing guided by siRNAs serves, among other purposes, as a genome defense system to suppress mobile genetic elements or invasive DNA (Dawe 2003; Schramke and Allshire 2003).

Using a genetic approach, we show here the existence of three small RNA-generating pathways with unique requirements in *Arabidopsis.* Plants with point mutations or insertions in several members of the *DCL* and *RDR* gene families were examined. The data indicate that plants genetically diversified several factors involved in formation of functionally distinct small RNAs.

## Results

### Genetic Requirements for miRNA Formation

**Figure 1:**
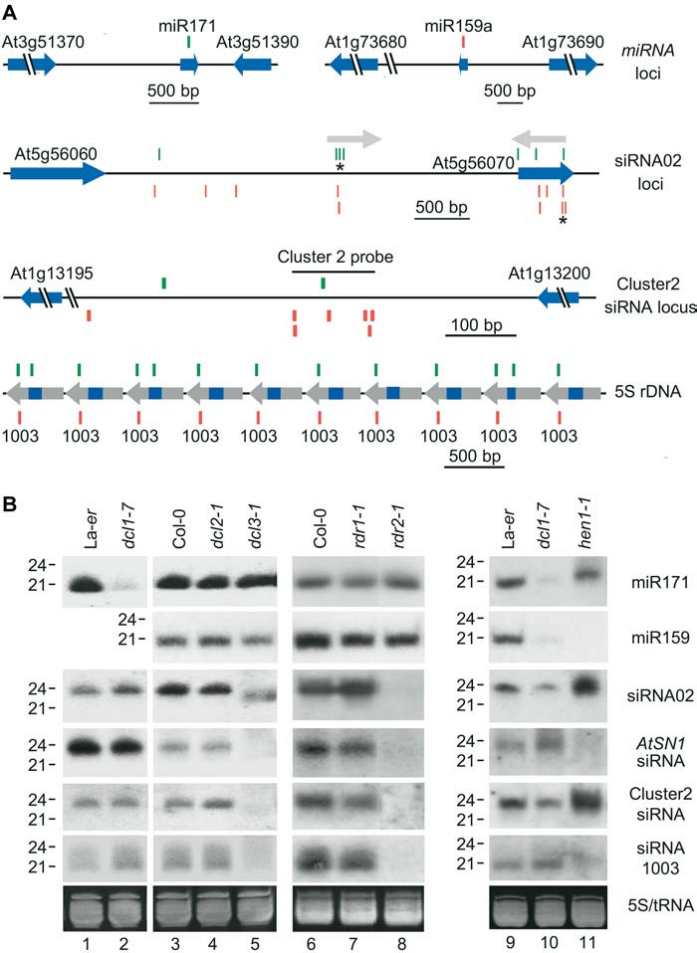
Genetic Requirements for miRNA and Endogenous siRNA Generation (A) miRNA genes and selected loci corresponding to three siRNAs or siRNA populations. Cloned small RNA sequenc-es are shown as green (sense orientation relative to the genome) or red (antisense orientation) bars. Protein-coding and miRNA genes are indicated by blue arrowheads. From top to bottom: miR-171 and miR-159a loci; siRNA02 loci, with each siRNA02 sequence indicated by an asterisk and the inverted duplication shown by the gray arrows; cluster2 siRNA locus; a segment of chromosome III showing 10 5S rDNA repeats (blue indicates 5S rRNA, gray indicates spacer) containing the siRNA1003 sequence. (B) Small RNA blot assays for miR-171, miR-159, and endogenous siRNAs. Ethid-ium bromide-stained gels (prior to transfer) in the zone corresponding to tRNA and 5S RNA are shown at the bottom. Each mutant is presented in a panel with the corresponding wild-type control (Col-0 or La-*er*).

At least two factors, DCL1 and HEN1 (HUA ENHANCER1), are involved in *Arabidopsis* miRNA formation. As shown for miR-171, miR-159 (Figure 1A), and several other miRNAs (Park et al. 2002; Reinhart et al. 2002), mutants with *dcl1* loss-of-function alleles lose most of their miRNA populations (Figure 1B). Plants with mutant *hen1* alleles either lose miRNAs or the apparent size of miRNAs is increased by one or more nucleotides (Park et al. 2002; Boutet et al. 2003) (Figure 1B). miRNA function to suppress target mRNAs is diminished in both *dcl1* and *hen1* mutants (Boutet et al. 2003; Kasschau et al. 2003; Xie et al. 2003). To determine whether other DCL or RDR proteins are required for miRNA formation in *Arabidopsis*, miR-171 and miR-159 were analyzed in four new mutants. The *dcl2-1* and *dcl3-1* mutants contained T-DNA insertions in *DCL2* (At3g03300) and *DCL3* (At3g43920) genes, respectively (Figure S1). In wild-type plants, *DCL2* and *DCL3* transcripts accumulated to detectable levels in inflorescence tissues, but not in leaves. The mutant *dcl2-1* and *dcl3-1* transcripts were not detected in either tissue type (Figure S1). The *rdr1-1* and *rdr2-1* mutants contained T-DNA insertions in *RDR1* (At1g14790) and *RDR2* (At4g11130), respectively (Figure S1). *RDR1* and *RDR2* transcripts accumulated in inflorescence tissue, but not leaves, of untreated wild-type plants (Figure S1). The *RDR1* transcript levels were elevated in salicylic acid (SA)-treated leaves, as shown previously (Yu et al. 2003), but *RDR2* transcript levels were not affected by SA (Figure S1). Both *rdr1-1* and *rdr2-1* transcripts were below the detection limit in the corresponding mutant plants. In addition, a mutant containing an insertion in the *RDR6* gene (also known as *SDE1/SGS2*; At3g49500) was analyzed in parallel with the *rdr1* and *rdr2* mutants. This *rdr6-1* mutant displayed a weak virus-susceptibility phenotype that was consistent with previously reported *sde1* and *sgs2* mutants (Mourrain et al. 2000; Dalmay et al. 2001). However, no differences in *RDR6* transcript levels were detected between wild-type and *rdr6-1* mutant plants (data not shown).

Accumulation of miR-171 and miR-159 was unaffected in the *dcl2* and *dcl3* mutants (see Figure 1B). This was in contrast to the low level or shifted mobility of miR-171 and miR-159 in *dcl1-7* and *hen1-1*, respectively (see Figure 1B). Similarly, accumulation of miR-171 and miR-159 was unaffected in *rdr1* and *rdr2* mutants.

### Composition of Endogenous siRNA Populations

**Figure 2:**
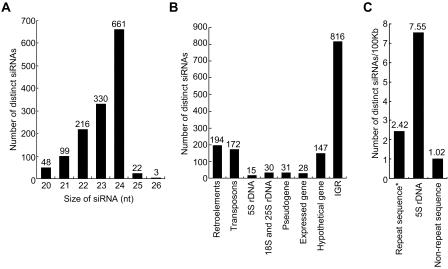
Endogenous siRNAs in *Arabidopsis* (A) Size distribution of endogenous siRNAs. (B) Distribution of distinct siRNAs in different sequence categories. (C) Density of siRNAs from highly repeated (mainly transposons and retroelements; the asterisk shows repeat sequences identified using RepeatMasker), 5S rDNA, and unique genomic sequence.

A library of cloned small RNAs from inflorescence tissues of Col-0 ecotype plants was partially sequenced and analyzed. Initial characterization of 125 of these sequences revealed that most of the clones corresponded to siRNA-like sequences (Llave et al. 2002a). A total of 1,368 distinct small RNAs, ranging in size between 20 and 26 nucleotides, were provisionally categorized here as siRNAs, with 24 nucleotides representing the most common size (Figure 2A; all sequences are available to view or download at http://cgrb.orst.edu/smallRNA/db/). The siRNA sequences were identified at 5,299 genomic loci (Table S1). Approximately 27% of endogenous siRNAs derived from transposon or retroelement sequences in the sense or antisense polarity (Figure 2B). Centromeric and pericentromeric siRNAs were common, which was partly due to the prevalence of transposons and retroelements at these sites. Forty-five small RNAs of sense and antisense polarity arose from highly repeated 5S, 18S, and 25S rDNA. While it is likely that some rDNA-derived sequences resulted from nonspecific breakdown of highly abundant rRNAs, some had specific genetic requirements and properties that were consistent with functional siRNAs (see below). Thirty-one siRNAs came from sequences annotated as psuedogenes and 147 from hypothetical or predicted genes (Figure 2B). Only 28 were identified as originating from genes that are known to be expressed (Figure 2B). The remaining 816 sequences mapped to loci that were collectively labeled as an IGR sequence. The IGR-derived siRNAs arose from unique sequences adjacent to known genes, inverted duplications, satellites, and other repeated sequences, although many of these may actually correspond to transposon or retroelement sequences that were not recognized by the search programs.

The frequency of unique siRNAs arising from highly repeated sequences (mainly transposons and retroelements), 5S rDNA repeats, and nonrepetitive sequence was calculated (Figure 2C). siRNAs in the library occurred at a frequency of 2.42 per 100 kb repetitive DNA, which was approximately 2.4-fold higher that the frequency of siRNAs from nonrepetitive sequence (1.02 per 100 kb). Based on the number of repeats in the most current version of the *Arabidopsis* genome sequence, unique siRNAs corresponding to 5S rDNA were identified at a frequency of 7.55 per 100 kb. These data indicate that siRNAs arise more frequently from highly repeat genome sequences.

### Genetic Requirements for Endogenous siRNA Formation

A set of four siRNAs or siRNA populations, representing the major categories identified in the library, were selected for genetic analysis. Twenty-six siRNAs corresponded to SINE retroelements, one of which (*AtSN1*) was selected for detailed analysis. *AtSN1*-derived siRNA formation requires *AGO4* (Zilberman et al. 2003) and *SDE4* (Hamilton et al. 2002). One siRNA (siRNA1003) originating from 5S rDNA was selected. The 5S rRNA genes occur in tandem arrays in chromosomes III, IV, and V, with the typical repeat unit (approximately 500 nucleotides) being composed of transcribed sequence (120 nucleotides) and flanking spacer sequences (Cloix et al. 2002; Mathieu et al. 2003). The siRNA1003 sequence was identified in the sense orientation within the spacer sequence in 202 repeats in chromosome III and four repeats in chromosome V (see Figure 1A). The cluster2 siRNA population from a 125-nucleotide IGR segment in chromosome I was represented by seven unique siRNAs in the library (see Figure 1A). Finally, the siRNA02 sequence corresponded to two loci separated by approximately 2.1 kb in chromosome V. One locus occurred in an IGR sequence, and the other within a hypothetical gene (At5g56070) of unknown function. The two siRNA02 loci occur in sequences that correspond to arms of an inverted duplication (see Figure 1A) (Llave et al. 2002a). The *AtSN1*, cluster2, and siRNA02 probes detected populations that accumulated as 24-nucleotide RNAs, while the siRNA1003 probe detected a population containing 21- to 24-nucleotide species (see Figure 1B).

The abundance of each siRNA population was decreased in the *dcl3-1* mutant, but not in the *dcl1-7* or *dcl2-1* mutants (see Figure 1B). This was in strict contrast to miR-171, miR-159 (see Figure 1B), and several other miRNAs tested (data not shown), which depended specifically on *DCL1*. Interestingly, weak signals corresponding to siRNA02, *AtSN1* siRNAs, and cluster2 siRNAs were detected in faster-migrating positions in the *dcl3-1* mutant (see Figure 1B). This may have resulted from exposure of siRNA precursors to alternate DCL activities in the absence of DCL3. Notably, both small and large siRNAs detected by the 5S rDNA-derived siRNA1003 probe were diminished in *dcl3-1* plants.

Each siRNA population was eliminated in the *rdr2-1* mutant, but not in the *rdr1-1* mutant (see Figure 1B). In preliminary experiments, each siRNA population was unaffected by the *rdr6-1* mutation, although these data should be interpreted cautiously because of the possibility that the *rdr6-1* allele is weak (data not shown). The endogenous siRNA requirement for *RDR2* contrasted with the miRNAs, which exhibited complete insensitivity to each of the *rdr* mutations tested (see Figure 1B). These data genetically identify DCL3 and RDR2 as components of an endogenous siRNA generating system that differs functionally from the miRNA-generating apparatus.

The HEN1 protein was implicated in post-transcriptional silencing of sense-, but not hairpin-forming, transgenes (Boutet et al. 2003). We tested the requirement of HEN1 for endogenous siRNA formation using the *hen1-1* mutant. Two of the siRNA populations, siRNA1003 and the *AtSN1*-siRNAs, were reduced to undetectable levels in *hen1-1* plants (see Figure 1B). The siRNA02 and cluster2 siRNAs, on the other hand, reproducibly accumulated to higher levels in *hen1-1* plants compared to wild-type La-*er* plants. Thus, each type of endogenous siRNA tested requires DCL3 and RDR2, but only the highly repeated 5S rDNA and retroelement-derived siRNAs require HEN1. In fact, the requirement for, or independence from, HEN1 was precisely the same as AGO4 at each of these loci (D. Zilberman and S. Jacobsen, unpublished data).

### Function of the Endogenous siRNA-Generating System

Two previous studies showed that SDE4 and AGO4 are required for *AtSN1* siRNA accumulation and methylation of cytosine positions at the *AtSN1* locus (Hamilton et al. 2002; Zilberman et al. 2003). In an *ago4* mutant, loss of *AtSN1* siRNA is associated with decreased histone H3K9 methylation (Zilberman et al. 2003). Cytosine methylation and increased histone H3K9 methylation are hallmarks of transcriptionally silent and heterochromatic DNA in plants and other organisms, and siRNAs may recruit chromatin modification complexes to specific loci (Grewal and Moazed 2003). To determine whether DCL3 and RDR2 catalyze formation of siRNAs that functionally interact with chromatin, cytosine methylation at *AtSN1* and 5S rDNA loci and methylation of H3K9 and H3K4 positions in *AtSN1* were examined in wild-type, *dcl3-1*, and *rdr2-1* plants. We also analyzed *AtSN1*-derived transcript levels to determine whether the mutations affected expression of the locus.

**Figure 3:**
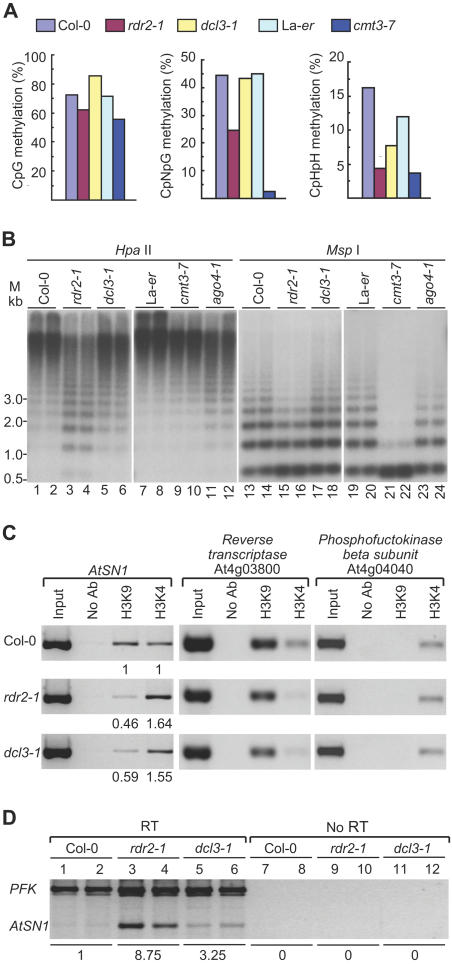
Effects of Mutations on *AtSN1* and 5S rDNA Chromatin Structure and Gene Expression (A) Analysis of CpG (left), CpNpG (center), and CpHpH (right) methylation in *AtSN1* by bisulfite sequencing of genomic DNA. (B) Blot analysis of 5S rDNA digested with methylation-sensitive restriction enzymes HpaII (left) and MspI (right). HpaII is sensitive to CpG and CpNpG methylation, whereas MspI is sensitive to only CpNpG methylation. Methylation is indicated by the ascending ladder, which corresponds to 5S rDNA multimers (monomer = approximately 0.5 kb). Duplicate samples from each plant were analyzed. (C) ChIP assays using antibodies against dimethyl-histone H3K9 and dimethyl-histone H3K4. Genomic DNA associated with immunoprecipitated chromatin was analyzed by semiquantitative PCR with primer pairs specific for *AtSN1*, retrotransposon reverse transcriptase (At4g03800) (internal control for H3K9 methylation), and PFK (At4g04040) (internal control for H3K4 methylation). The PCR products were quantitated and compared against the respective internal controls, and the relative H3K4 and H3K9 methylation levels were expressed relative to that in Col-0 (arbitrarily set to 1.00). (D) Detection of *AtSN1*-specific transcripts by semiquantitative RT-PCR. Primers specific for PFK transcripts were used as the internal control. A parallel set of reactions without addition of reverse transcriptase (RT) was run as a quality control for genomic DNA contamination. The PCR products were normalized relative to PFK, and the expression levels were calculated relative to that in Col-0 (arbitrarily set to 1.00).

Consistent with previous reports (Hamilton et al. 2002; Zilberman et al. 2003), bisulfite sequencing of *AtSN1* genomic DNA revealed extensive CpG (72.0%), CpNpG (43.1%), and asymmetric CpHpH (16.3%) methylation in Col-0 wild-type plants (Figure 3A; Table S2). In the *rdr2-1* mutant, CpNpG and CpHpH methylation was reduced to 24.6% and 4.5%, respectively. Only a slight reduction in CpG methylation was detected in *rdr2-1* plants (Figure 3A). This methylation pattern was similar to that detected in mutants lacking CHROMOMETHYLASE3 (*cmt3-7*; Figure 3A), which is necessary for efficient methylation of *AtSN1* at non-CpG sites, and in a mutant lacking AGO4 (Zilberman et al. 2003). In the *dcl3-1* mutant, however, cytosine methylation was decreased only at asymmetric sites, while CpG and CpNpG methylation was similar to that of wild-type plants (Figure 3A).

Because of the number of 5S rDNA repeats, analysis of cytosine methylation was done using restriction enzymes HpaII or MspI and DNA blot assays. Sensitivity to HpaII indicates lack of methylation at CpG or CpNpG sites (or both), whereas sensitivity to MspI indicates lack of methylation at only CpNpG sites. In wild-type Col-0 and La-*er* plants, 5S rDNA loci were heavily methylated at CpG+CpNpG sites, as shown by detection of only high molecular weight forms using HpaII, and partially methylated at CpNpG as shown using MspI (Figure 3B). In *rdr2-1* plants, methylation was partially lost at CpNpG sites (increased MspI sensitivity; Figure 3B, lanes 15–16), although to a lesser degree than in *cmt3-7* plants (Figure 3B, lanes 21–22). Methylation detected by HpaII sensitivity was partially lost in the *rdr2-1* mutant (Figure 3B, lanes 3–4), which was most likely due to loss of CpG methylation. Loss of only CpNpG methylation in *rdr2-1* plants would not account for the increased sensitivity to HpaII, as HpaII sensitivity in *cmt3-7* plants (lacking nearly all CpNpG methylation) was unaffected (Figure 3B, lanes 9–10). Sensitivity of 5S rDNA sites to HpaII and MspI in *dcl3-1* plants was only slightly increased (Figure 3B, lanes 5–6 and 17–18). In the *ago4-1* mutant, CpG methylation was partially lost as revealed by increased sensitivity to HpaII (Figure 3B, lanes 11–12).

Chromatin immunoprecipitation (ChIP) assays were used to detect changes in H3K4 and H3K9 methylation at *AtSN1* in *rdr2-1* and *dcl3-1* mutant lines. Loci containing genes encoding a retrotransposon reverse transcriptase and phosphofructokinase β subunit (PFK) were used as positive controls for sequences associated primarily with K9- and K4-methylated histone H3, respectively (Gendrel et al. 2002). At *AtSN1*, decreased levels of histone H3K9 methylation were detected in both *rdr2-1* and *dcl3-1* mutants (see Figure 3C). This was accompanied by a slight increase in H3K4 methylation (see Figure 3C). The extent to which H3 methylation changed was greater in *rdr2-1* relative to *dcl3-1* plants. Little or no change in H3K4 and H3K9 methylation was detected at the control loci. In addition, no changes in H3K4 or H3K9 methylation were detected at *AtSN1* in *cmt3-7* plants (data not shown). The changes in H3 methylation shown here are similar to those at several heterochromatic or silenced loci in *ago4* mutant plants (Zilberman et al. 2003).

The level of *AtSN1*-derived transcripts was measured in *rdr2-1* and *dcl3-1* mutant plants and compared against the level of PFK transcript using semiquantitative RT-PCR. As shown in Figure 3D, relatively low levels of *AtSN1* transcripts were detected in wild-type Col-0 plants. However, the normalized level of *AtSN1* transcripts was over 8- and 3-fold higher in *rdr2-1* and *dcl3-1* mutant plants, respectively, compared to wild-type plants. Therefore, loss of siRNA-forming capability correlated with loss of heterochromatic marks and elevated transcript levels at an endogenous locus that is normally silenced at the chromatin level.

**Figure 4:**
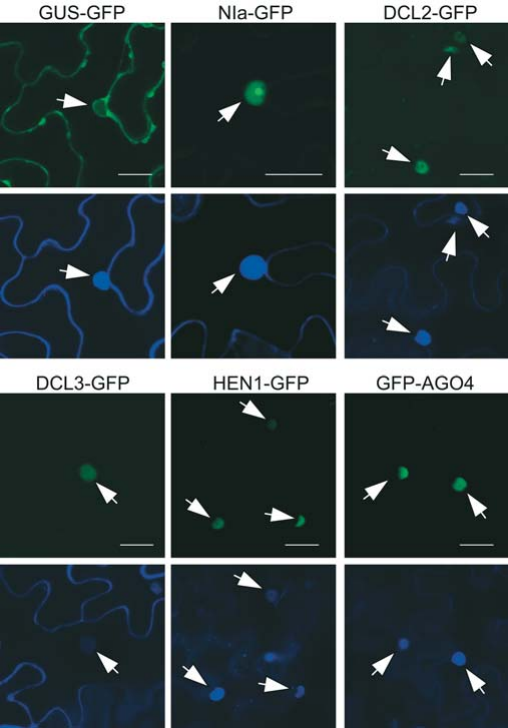
Subcellular Localization of GFP Fusion Proteins Pairwise presentation of confocal microscopic images showing GFP fluorescence (top) and DAPI fluorescence (bottom) in N. benthamiana expressing the indicated GFP fusion proteins. Arrowheads indicate the location of nuclei. Note that the GUS–GFP control protein accumulates in cytoplasm at the cell periphery and immediately surrounding nuclei, while the NIa–GFP control protein accumulates in nuclei. Scale bar = 25μm.

Given that RDR2, DCL3, and AGO4 are involved in chromatin-associated events and that HEN1 is required for accumulation of certain endogenous siRNAs associated with chromatin modification, it was hypothesized that each of these proteins accumulates in the nucleus. The presence of nuclear transport signals in each protein was tested by transient expression and analysis of green fluorescent protein (GFP) fusions in a heterologous plant, Nicotiana benthamiana, using an *Agrobacterium* infiltration assay. Subcellular accumulation sites for these proteins were compared to those of β-glucurodinase (GUS)–GFP (cytosolic control) and nuclear inclusion a protein (NIa)–GFP (nuclear control). The DCL3–GFP, HEN1–GFP, and GFP–AGO4 fusion proteins were detected exclusively in the nucleus (Figure 4; Figure S2), indicating that DCL3, HEN1, and AGO4 possess independent nuclear transport capability. Subcellular localization experiments with RDR2–GFP and GFP–RDR2 fusion proteins, however, were inconclusive due to low expression levels and protein instability (data not shown).

### Genetic Requirements for Virus-Derived siRNA Formation

The involvement of DCL1, DCL2, and DCL3 in siRNA formation in response to infection by three dissimilar RNA viruses was tested using the *dcl* mutant series. Two of the viruses, a GFP-tagged version of turnip mosaic virus (TuMV–GFP) and turnip crinkle virus (TCV), infect *Arabidopsis* systemically and cause moderate to severe disease symptoms. The third virus, cucumber mosaic virus strain Y (CMV-Y), infects plants systemically, but causes only mild symptoms. Wild-type (Col-0 and La-*er*) and mutant plants were inoculated on rosette leaves, and upper, noninoculated tissue (cauline leaves and inflorescences) was analyzed for virus-specific siRNAs at 7 and 14 d post-inoculation (dpi).

**Figure 5:**
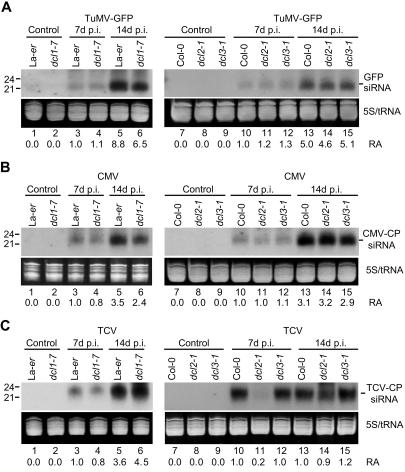
Genetic Requirements for DCLs in Viral siRNA Generation Blot analysis of viral siRNA. Systemic tissue samples were analyzed at the indicated time points from parental and mutant lines that were infected with TuMV–GFP (A), CMV-Y (B), and TCV (C). RNA blots were analyzed using virus-specific probes to detect siRNAs. Ethidium bromide-stained gels in the zone corresponding to tRNA and 5S RNA are shown. Relative accumulation (RA) of siRNAs is indicated at the bottom of each panel, with the level measured in infected control plants (Col-0 or La-*er*, depending on the mutant) at 7 dpi arbitrarily set to 1.0.

**Figure 6:**
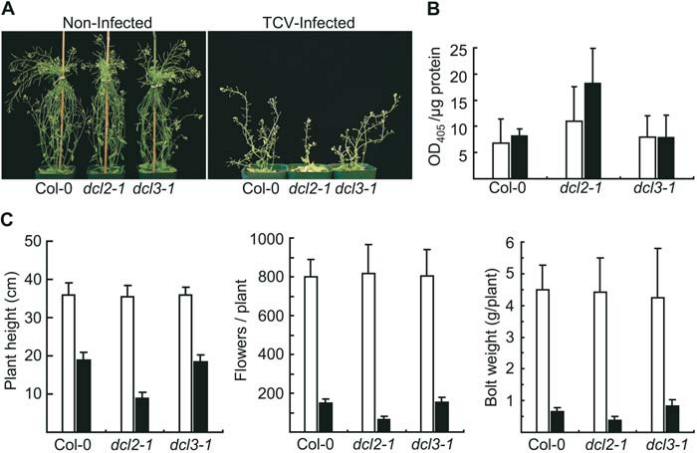
Altered Susceptibility to TCV Infection in *dcl2-1* Mutant Plants (A) Noninfected control (left) and TCV-infected (right) Col-0, *dcl2-1*, and *dcl3-1* plants at 14 dpi. (B) TCV accumulation, as measured by ELISA, in the systemic tissues of infected wild-type and mutant plants at 7 dpi (open bars) and 14 dpi (filled bars). (C) Plant height (left), number of flowers/plant (center), and fresh weight of bolt tissue (right) were measured at 14 dpi in noninfected (open bars) and infected (filled bars) plants (*n* = 9).

Viral siRNAs were detected in systemic tissues from wild-type plants at both timepoints (Figure 5A–5C, lanes 3, 5, 10, and 13), with siRNA levels generally higher at 14 dpi. In TuMV- and CMV-infected *dcl1-7*, *dcl2-1*, and *dcl3-1* mutant plants, siRNAs accumulated to levels that were similar to those in infected wild-type plants at 7 and 14 dpi (Figures 5A and 5B). TuMV and CMV titers and symptom phenotypes in the three mutants were indistinguishable from those in their respective parents (data not shown). Similarly, in TCV-infected *dcl1-7* and *dcl3-1* plants, viral siRNA levels, virus titer, and symptom severity were essentially the same as in wild-type plants (Figure 5C; Figure 6A and 6B; data not shown).

In contrast, TCV-derived siRNAs accumulated to levels that were 5-fold lower in *dcl2-1* plants compared to wild-type plants at 7 dpi (see Figure 5C, lanes 10–11). This was a transient deficit, as TCV siRNA levels rebounded to near wild-type levels by 14 dpi (see Figure 5C, lanes 13–14). The slow accumulation of siRNAs was not due to lack of TCV replication or movement in the tissues analyzed, as TCV titer in the *dcl2-1* mutant was similar to (7 dpi) or significantly higher than (*p* < 0.05, 14 dpi) the titers in wild-type plants (Figure 6B). Additionally, TCV-induced disease was more severe in *dcl2-1* plants, as plant height, fresh weight of bolts, and number of flowers in infected *dcl2-1* plants were each significantly (*p* < 0.01 for plant height and flower number; *p* < 0.05 for weight of bolts) lower compared to infected wild-type plants (Figure 6A and 6C). Therefore, DCL2 functions as a component of the antiviral silencing response in TCV-infected plants.

The DCL2–GFP fusion protein accumulated predominantly in the nucleus of N. benthamiana cells in the transient assay system, although some cytosolic localization was also detected (see Figure 4). Thus, DCL1 (Papp et al. 2003), DCL2, and DCL3 each have nuclear transport activity.

## Discussion

### Genetic Diversification of Small RNA-Generating Systems in Plants

We show here that *Arabidopsis* has at least three systems to generate distinct classes of endogenous or virus-induced small RNAs and that these are associated with specialized regulatory or defensive functions. First, the miRNA-generating system requires DCL1, as shown previously (Park et al. 2002; Reinhart et al. 2002), but none of the RDR proteins tested. In principle, there should be no requirement for an RDR activity during miRNA biogenesis, as the DCL1 substrate is formed directly as a result of DNA-based transcription. DCL1 likely functions in the nucleus (Papp et al. 2003). It also functions, either directly or indirectly, with HEN1, which may confer substrate specificity, processing accuracy, or catalytic function.

The second system requires DCL3 and RDR2 and generates endogenous siRNAs primarily of the large-sized (approximately 24 nucleotides) class. While DCL3 undoubtedly functions as the ribonuclease to process dsRNA precursors, RDR2 presumably functions as a polymerase to form dsRNA molecules de novo using templates resulting from transcription of DNA. At some loci, however, RDR2 may be unnecessary as a catalytic subunit, but rather contribute to the formation or stability of a complex that contains active DCL3. This could be the case at some sites, such as the siRNA02 locus, that contain inverted duplications and that may form transcripts with extensive dsRNA structure. Interestingly, accumulation of siRNAs specific to a hairpin construct was shown to be RdRp dependent in fission yeast (Schramke and Allshire 2003). At some loci, this system appears to interface with AGO4, HEN1, and SDE4.

The third system functions in antiviral defense and involves DCL2. Loss of this system was specifically detected in TCV-infected *dcl2-1* plants, which exhibited delayed viral siRNA accumulation and increased susceptibility and sensitivity. However, there are several reasons to suspect that multiple antiviral, siRNA-generating systems exist. siRNAs triggered by TCV were not eliminated in *dcl2-1* plants, but rather siRNA accumulation was delayed. Although this could be due to incomplete loss of DCL2 function in the mutant, it could also reflect the existence of secondary or redundant DCL activities. Among the three viruses tested, two were unaffected by the *dcl2-1* mutation. This strongly implies the existence of one or more other siRNA-generating activities with unique or redundant antiviral specificity. Further, the DCL2-dependent system may have functions in addition to those associated with antiviral defense. The DCL2–GFP fusion protein was detected primarily in the nucleus, whereas TCV replicates and accumulates outside of the nucleus. Experiments to determine the genetic requirements for RDR1 and RDR2 during antiviral silencing against the three viruses were inconclusive, again possibly the result of functional redundancies or the presence of confounding viral RdRp activities (Ahlquist 2002). Mourrain et al. (2000), on the other hand, showed that *rdr6* (*sde1/sgs2*) mutants were deficient in CMV-induced silencing. Additionally, Yu et al. (2003) showed that *RDR1* contributed to defense against tobamoviruses.

Tang et al. (2003) identified two siRNA-generating DCL activities in wheat-germ extracts. These were detected using dsRNA as a substrate. Although monocots contain a *DCL* gene family, the members do not correlate one-for-one with those in *Arabidopsis* (Z. Xie and J. Carrington, unpublished data). Further study is required to correlate the DCL activities from wheat germ with those in *Arabidopsis.*

The degree of genetic diversification of the DCL family in plants is in contrast to the situation in animals. Caenorhabditis elegans and human, for example, contain only one DICER (Grishok et al. 2001; Ketting et al. 2001; Knight and Bass 2001; Provost et al. 2002; Zhang et al. 2002), even though both possess miRNA and siRNA functions. Thus, whereas plants diversified and functionally specialized DCL family members during evolution, animals evolved functionally distinct small RNA systems around one or relatively few DICER activities. Animals, however, evolved relatively large AGO-related families (Carmell et al. 2002), and these may provide modules for functional specialization.

### Roles of Endogenous siRNA-Generating Systems in Plants

Both DCL3 and RDR2 cooperate with AGO4, and possibly also with SDE4 and HEN1, at the *AtSN1* locus to initiate or maintain a heterochromatic state (Hamilton et al. 2002; Zilberman et al. 2003). Loss of DCL3, RDR2, and AGO4 factors correlates with loss of DNA methylation and histone H3K9 methylation. Interestingly, these factors are also necessary for silencing triggered de novo during the transformation process using transgenic *FWA* (Chan et al. 2004). Silencing of *FWA* is due to cytosine methylation of a region in the promoter that contains direct repeats (Soppe et al. 2000). The effect of the *rdr2-1* mutation on chromatin structure and gene silencing of *AtSN1* and *FWA* was generally stronger than the effect of the *dcl3-1* mutation. This may be explained by the presence of residual siRNAs formed by another DCL activity in the *dcl3* mutant (see Figure 1B). The picture that emerges from these and other results shows that DCL3 and RDR2 function as components of an endogenous siRNA-generating system and that the resulting siRNAs may guide chromatin modification events through effector complexes containing AGO4. Given that AGO proteins are components of RISCs that catalyze sequence-specific RNA degradation (Carmell et al. 2002) and that different AGO proteins have DNA- or RNA-binding activities (Lingel et al. 2003; Song et al. 2003; Yan et al. 2003), it seems reasonable to speculate that AGO4 engages a chromatin-associated RISC-like complex and interacts with nuclear siRNAs or target sequences. But unlike RNAi events in the cytoplasm, chromatin-associated complexes likely interact with DNA methyltransferase and histone methyltransferase systems. RdDM can occur at CpG and non-CpG sites, but maintenance of non-CpG methylation after DNA replication may generally require the continued activity of the siRNA-guided complex (Luff et al. 1999; Jones et al. 2001; Aufsatz et al. 2002). Methylation at CpG sites, in contrast, can be maintained by template-driven methylation on hemimethylated products of DNA replication, which explains why CpG methylation frequently persists in subsequent generations after one or more silencing factors or trigger loci are lost.

Accumulation of siRNA from endogenous loci and transgenes does not necessarily require AGO4 (D. Zilberman and S. Jacobsen, unpublished data), suggesting that AGO4 acts downstream of siRNA formation to direct DNA methylation. Losses of AGO4 and HEN1 have nearly identical effects on all siRNAs tested, possibly because HEN1 and AGO4 affect a similar point in the pathway. If AGO4 and HEN1 function downstream of siRNA formation, why do siRNAs derived from some sites (*AtSN1* and 5S rDNA) accumulate to such low levels in *ago4* and *hen1* mutants? One possibility is that heterochromatic marks (DNA and H3K9 methylation) and associated factors serve to recruit RDR2, DCL3, or both to specific sites on chromatin, thus establishing a reinforcement loop. Loss of heterochromatin in an *ago4* mutant, for example, would result in failure to recruit the siRNA-generating enzymes to transcripts originating from a target locus and, therefore, the absence of siRNAs. This hypothesis, however, does not hold for some other siRNA-generating sites, such as those that yield cluster2 siRNAs and siRNA02. Accumulation of siRNAs from these sites is unaffected or even enhanced in *ago4* and *hen1* mutants. In wild-type plants, these loci are both hypomethylated at CpG and non-CpG sites and are associated with histone H3 that largely lacks K9 methylation (data not shown). The siRNAs formed from these loci clearly require RDR2 and DCL3, but they appear not to affect chromatin structure. These siRNAs may be sequestered elsewhere in the cell and unable to interact with chromatin or chromatin-associated factors.

The spectrum of naturally occurring siRNAs in *Arabidopsis* is informative about the roles of these molecules in genome maintenance, genome expression, and defense. The fact that siRNAs from highly repeated sequences, largely retroelements and transposons, are overrepresented compared to unique genome sequences suggests that sequence duplication events are sensed and dealt with through RNA-guided formation of heterochromatin. This is frequently discussed within the context of genome defense, whereby suppression of mobile DNA promotes genome stability (Plasterk 2002; Dawe 2003). Indeed, loss of heterochromatin is often associated with increased activity of transposons and retroelements (Hirochika et al. 2000; Miura et al. 2001; Singer et al. 2001; Gendrel et al. 2002). However, it should be appreciated that these and other repeated sequences might also serve as *cis*-active, epigenetic regulatory modules if positioned near or within functional genes (Kinoshita et al. 2004). The rapidly expanding number of examples, such as vernalization (Bastow and Dean 2003), of cellular memory conditioned by epigenetic events hint that siRNA-directed processes may be embedded broadly as a regulatory mechanism during growth and development (Goodrich and Tweedie 2002).

## Materials and Methods

### 

#### Plant materials

All plants were grown under standard greenhouse conditions. The *dcl1-*7, *hen1-1*, *cmt3-7*, and *ago4-1* mutant lines were described previously (Cao and Jacobsen 2002; Golden et al. 2002; Park et al. 2002; Zilberman et al. 2003). Other mutant lines were obtained from the Salk Institute Genome Analysis Laboratory (SIGnAL, La Jolla, California, United States) and Torrey Mesa Research Institute (now a subsidiary of Syngenta, Basel, Switzerland). *dcl2-1* has a T-DNA insertion within predicted intron 9 (after nucleotide 2,842 from ATG of the genomic DNA) of *DCL2* (At3g03300). *dcl3-1* has a T-DNA insertion within predicted exon 7 of *DCL3* (At3g43920) at a point 2,136 nucleotides beyond the ATG in genomic DNA. This introduces four codons after the serine 288 codon, followed by a premature stop codon. *rdr1-1* has a T-DNA insertion within predicted exon 1 after nucleotide 2,366 beyond the ATG of *RDR1* (At1g14790). *rdr2-1* has a T-DNA insertion within predicted exon 1 (in front of nucleotide 316 from the ATG) of *RDR2* (At4g11130). *rdr6-1* has a T-DNA insertion within predicted exon 2 (in front of nucleotide 3,977 from ATG of the genomic DNA) of *RDR6* (also known as *SDE1/SGS2*; At3g49500). Each insertion line was backcrossed twice to Col-0 and brought to homozygosity. Additional information about the insertion lines are provided in the supplemental online materials.

For analysis of each insertion mutant, Col-0 was the wild-type control plant. For *dcl1-7*, *hen1-1*, *ago4-1*, and *cmt3-7* mutants, La-*er* was the wild-type control.

#### RNA blot analysis

Extraction of low- and high-molecular weight RNAs and blot assays were done as described previously (Llave et al. 2002a). Low-molecular weight RNA (20 μg) from *Arabidopsis* inflo-rescence tissue was used for miRNA and endogenous siRNA analysis. Probes for miR-171 and *AtSN1*-siRNA analysis were described previously (Llave et al. 2002b; Zilberman et al. 2003). miR-159 was detected using an end-labeled DNA oligonucleotide AS-159 (5′-TAGAGCTCCCTTCAATCCAAA-3′). siRNA02 and siRNA1003 were detected using the end-labeled DNA oligonucleotides AS-02 (5′-GTTGACCAGTCCGCCAGCCGAT-3′) and AS-1003 (5′-ATGCCAAGTTTGGCCTCACGGTCT-3′), respectively. The probe for cluster2 siRNAs was a random primer-labeled fragment spanning a 235-nucleotide IGR of chromosome I (nucleotides 4,506,544–4,506,778) (see Figure 1A) and was amplified from genomic DNA using primers AS-285 (5′-TTGCTGATTTGTATTTTATGCAT-3′) and S-786 (5′-CTTTTTCAAACCATAAACCAGAAA-3′).

#### Analysis of DNA and histone methylation

Cytosine methylation was analyzed by bisulfite sequencing of genomic DNA or by DNA blot assay following digestion with methylation-sensitive restriction endonucleases, as described elsewhere (Jacobsen et al. 2000; Zilberman et al. 2003). The region of *AtSN1* analyzed (chromosome III, nucleotides 15,805,617–15,805,773) was treated with sodium bisulfite and amplified using primers *AtSN1*-BS1 (5′-GTTGTATAAGTTTAGTTTTAATTTTAYGGATYAGTATTAATTT-3′) and *AtSN1*-BS2 (5′-CAATATACRATCCAAAAAACARTTATTAAAATAATATCTTAA-3′). At least 18 independent clones were sequenced for each genotype.

ChIP assays were done using antibodies specific for dimethyl-histone H3K4 (Upstate Biotechnology, Lake Placid, New York, United States) or dimethyl-histone H3K9 (kindly provided by T. Jenuwein, Research Institute of Molecular Pathology, Vienna, Austria) as described elsewhere (Gendrel et al. 2002). Methylation of H3K4 and H3K9 at *AtSN1* in wild-type Col-0 and *rdr2-1* and *dcl3-1* mutants was measured relative to that at internal control loci, At4g04040 and At4g03800. The data were then normalized against the values measured in Col-0.

#### Analysis of GFP fusion proteins

The *35S:DCL3–GFP* construct contained the *DCL3* coding region fused to GFP coding sequence, flanked by the cauliflower mosaic virus (CaMV) 35S promoter and terminator sequences. The expression cassette was cloned in pSLJ755I5. All other GFP fusion constructs were made by cloning the coding sequence into pGWB5 (for C-terminal GFP) or pGWB6 (for N-terminal GFP), a set of gateway-compatible binary vectors designed for 35S promoter-driven expression of GFP fusion proteins (kindly provided by T. Nakagawa, Shimane University, Izumo, Japan). Cloning using gateway vectors was done using reagents and protocols from Invitrogen (Carlsbad, California, United States). Constructs were introduced into Agrobacterium tumefaciens strain GV2260 and expressed in N. benthamiana leaves as described previously (Johansen and Carrington 2001). Fusion proteins were detected by confocal microscopy and immunoblot assay using a monoclonal antibody against GFP (Roche, Basel, Switzerland).

#### Virus infection assays

Wild-type and mutant *Arabidopsis* plants (approximately 4 wk old, prior to bolting) were infected with TuMV–GFP, CMV-Y, and TCV as described previously (Whitham et al. 2000; Lellis et al. 2002). At 7 and 14 dpi, systemic tissues consisting of inflorescences and cauline leaves were harvested for ELISA and RNA blot assays. Antibodies used for TuMV and TCV ELISAs were as described previously (Lellis et al. 2002).

#### Computational methods

Computational identification of repeat sequences, including transposons and retroelements, in the *Arabidopsis* genome was done using RepeatMasker (http://ftp.genome.washington.edu/RM/RepeatMasker.html) and Repbase (http://www.girinst.org/index.html).

Further information about *Arabidopsis* siRNAs and miRNAs, including those that were analyzed in this work, can be found in the *Arabidopsis* Small RNA Project database (http://cgrb.orst.edu/smallRNA/db/).

## Supporting Information

Figure S1 *DCL* and *RDR* Mutant Lines (A) Exon (bars)/intron (lines) organization of the *Arabidopsis DCL* and *RDR* genes and location of T-DNA insertion sites in mutant lines.
(B) RNA blot analysis (20 μg of total RNA) for *DCL2* and *DCL3* mRNA in Col-0 and the respective mutants. DNA fragments corresponding to nucleotides 2,652–3,292 of the *DCL2* open reading frame and nucleotides 2,805–3,571 of the *DCL3* open reading frame were used as hybridization probes. As a control, the blots were stripped and hybridized with a β-tubulin-specific probe (Kasschau et al. 2003).
(C) RNA blot analysis (10 μg of total RNA) for *RDR1* and *RDR2* mRNA in Col-0 and the respective mutants. DNA fragments corresponding to nucleotides 2,900–3,300 of the *RDR1* open reading frame and nucleotides 10–271 of the *RDR2* open reading frame were used as gene-specific probes. RNA samples from SA-treated leaf tissues were also included in the analysis.
(5.9 MB EPS).

Figure S2 Immunoblot Analysis of GFP Fusion Proteins The 35S promoter-driven GFP fusion constructs were transiently expressed in N. benthamiana using an *Agrobacterium*-injection procedure. Leaf tissue from injected zones was excised at 2 dpi for immunoblot assay using a monoclonal antibody against GFP and confocal microscopy (see Figure 4). An arrow indicates the position of predicted full-sized fusion protein.
(10.8 MB EPS).

Table S1 Cloned siRNA Loci in the *Arabidopsis* Genome (25 KB DOC).

Table S2 Cytosine Methylation of *Arabidopsis AtSN1*
(24 KB DOC).

### Accession Numbers

The GenBank (http://www.ncbi.nlm.nih.gov/Genbank/) accession numbers for the entities discussed in this paper are At1g14790 (NM_101348), At3g03300 (NM_111200), At3g43920 (NM_114260), At3g49500 (NM_114810), At4g11130 (NM_117183), chromosome I (NC_003070.3), chromosome III (NC_003074.4), and siRNA02 (AF501743).

The SAIL (formerly Garlic) (http://signal.salk.edu/cgi-bin/tdnaexpress) accession numbers for the T-DNA insertion lines discussed in this paper are *rdr1-1* (SAIL_672F11), *rdr2-1* (SAIL_1277H08), and *rdr6-1* (SAIL_388H03).

The SIGnAL database (http://signal.salk.edu/) accession numbers for the T-DNA insertion lines discussed in the paper are *dcl2-1* (SALK_064627) and *dcl3-1* (SALK_005512).

## Abbreviations

AGO: ARGONAUTE
*AtSN1*: Arabidopsis thaliana short interspersed element 1
CaMV: cauliflower mosaic virus
ChIP: chromatin immunoprecipitation
CMT3: CHROMOMETHYLASE3
CMV: cucumber mosaic virus*;* DCL
dpi: days post-inoculation
dsRNA: double-stranded RNA
GFP: green fluorescent protein
GUS: β-glucurodinase
H3K4: histone H3 at the lysine 4
H3K9: histone H3 at the lysine 9
HEN1: HUA ENHANCER 1
IGR: intergenic region
miRNA: microRNA
NIa: nuclear inclusion a protein
PFK: phosphofructokinase β subunit
RdDM: RNA-directed DNA methylation
*RDR*: RNA-dependent RNA polymerase gene
RdRp: RNA-dependent RNA polymerases
RISC: RNA-induced silencing complex
RNAi: RNA interference
RT: reverse transcriptase
SA: salicylic acid
siRNA: short interfering RNA
TCV: turnip crinkle virus
TuMV: turnip mosaic virus
