# Supplementary material for: Genetic and Functional Diversification of Small RNA Pathways in Plants
Source: PLoS Biol. 2004 Feb 24;2(5):e104. doi: 10.1371/journal.pbio.0020104 (PMC350667; doi:10.1371/journal.pbio.0020104)
Supplement: Supplementary file 1 [file pbio.0020104.sd001.doc]

Table S1. The siRNA loci in Arabidopsis Genome[a]

Chromosome	Total Loci	Retroelements[b]		Transposons[b]		5S rDNA		Pseudogene		Expressed gene		Hypothetical gene		IGR
		S[c]	AS[c]	S	AS	S	AS	S	AS	S	AS	S	AS	
1	1030	114	182	67	208	0	1	2	30	5	1	47	23	350
2	791	63	167	72	160	0	0	2	4	1	3	45	41	233
3	1487	115	187	72	167	215	406	2	2	5	8	20	15	273
4	844	79	183	46	125	28	39	2	3	0	1	18	17	303
5	1147	99	195	57	215	21	38	0	1	3	4	10	24	480
TOTAL	5299	470	914	314	875	264	484	8	40	14	17	140	120	1639

[a]siRNAs that hit 18S and 25S rRNA loci are not included because the number of 18S and 25S rRNA loci is not given in the current genome sequence version.

[b]Predicted using RepeatMasker program as indicated in the text.

[c]S: Sense; AS: Antisense.
