# Supplementary material for: Genetic and Functional Diversification of Small RNA Pathways in Plants
Source: PLoS Biol. 2004 Feb 24;2(5):e104. doi: 10.1371/journal.pbio.0020104 (PMC350667; doi:10.1371/journal.pbio.0020104)
Supplement: Supplementary file 2 [file pbio.0020104.sd002.doc]

Table S2. Cytosine Methylation of Arabidopsis *AtSN1*

Genotype[a]	CpG[b]		CpNpG		CpHpH[c]	
Col-0	121/168[d]	(72.0%)	94/218	(43.1%)	287/1759	(16.3%)
rdr2-1	64/104	(61.5%)	35/142	(24.6%)	52/1168	(4.5%)
dcl3-1	76/89	(85.4%)	51/119	(42.9%)	70/915	(7.7%)
La-*er*	56/78	(71.8%)	45/100	(45.0%)	90/757	(11.9%)
cmt3-7	40/72	(55.6%)	2/88	(2.3%)	24/679	(3.5%)

[a]Eighteen to forty-seven clones were sequenced for each genotype.

[b]CpGpG sites are counted as CpG sites, not as CpNpG.

[c]H = A, T, or C.

[d]Number of methylated cytosines/total number of sites analyzed.
